# Pediatric Central Retinal Artery Occlusion Attributed to a Patent Ductus Arteriosus

**DOI:** 10.7759/cureus.61083

**Published:** 2024-05-25

**Authors:** Mikes N Glynatsis, Marina Economou, Kalliopi Papadopoulou, Ioanna Mylona

**Affiliations:** 1 Department of Ophthalmology, ‘Hippokration’ General Hospital of Thessaloniki, Thessaloniki, GRC; 2 1st Department of Pediatrics, ‘Hippokration’ General Hospital of Thessaloniki, Aristotle University, Thessaloniki, GRC; 3 Department of Ophthalmology, General Hospital of Serres, Serres, GRC

**Keywords:** transthoracic echocardiogram, pediatric, patent ductus arteriosus, artery occlusion, central retinal artery

## Abstract

This report presents a rare case of a central retinal artery occlusion in an eight-year-old girl attributed to an undiagnosed patent ductus arteriosus (PDA). Despite intensive treatment, the patient’s eyesight failed to improve. Cases of central retinal artery occlusion may occur in patients with undiagnosed, small PDA, with only symptomatic treatment being available.

## Introduction

Pediatric central retinal artery occlusion is an extremely rare clinical syndrome that may lead to permanent loss of vision if the patient does not respond to symptomatic treatment. We present one such case of an eight-year-old girl with poor outcome associated with an undiagnosed patent ductus arteriosus.

Patent ductus arteriosus (PDA) refers to the pathological failure of the patent arterial duct, that diverts placental oxygenated blood from the pulmonary artery into the aorta by-passing lungs, to close [1]. While PDA is a common disorder in preterm infants, occurring in up to 60% of infants born at <29 weeks gestational age, there is a considerably smaller incidence of only 57 out of 100,000 full-term neonates [2]. PDA is associated with mortality and severe morbidity, correlated with the size of the lesion [1], with larger lesions presenting with pulmonary hemorrhage, or with cardiomegaly and signs of pulmonary edema, as well as respiratory failure and/or arterial hypotension requiring vasopressor treatment. Smaller defects remaining asymptomatic and undetected until well into maturity. Rare cases of thromboembolism attributed to abnormal blood flow from a PDA have been reported in the past, in neonates [3] and in adults [4,5].

Central retinal artery occlusion (CRAO) in children is an extremely rare clinical syndrome, characterized by rapid onset of vision loss in the affected eye, which may become permanent. In most instances, there is a discreet etiology uncovered after the onset of the event, with hypercoagulable states and emboli being the most frequent causes [6], although cases with no apparent cause are also rarely seen [7-9]. The incidence of retinal arterial occlusion in patients under the age of 30 years has been estimated at less than one in 50,000 outpatients [6]. We present a case of a healthy eight-year-old girl with a previously undetected PDA, which unfortunately failed to respond adequately to symptomatic treatment.

## Case presentation

The patient is an eight-year-old girl who presented to the Emergency Department of our hospital with acute onset of vision loss in her left eye. She demonstrates a healthy general physical status, without specified complaints and no history of thrombophilia, recent infection, or circulatory disorders. From past history, the child was born after a full-term pregnancy. She was referred for two unexplained fainting episodes; the standard evaluation for syncope with physical examination and electrocardiography did not reveal a cause and the patient was followed by a pediatric neurologist one year ago; a small cerebellar hemangioma was found but not linked to the episodes. Those were classified as neutrally mediated syncope episodes and not followed through as the patient did not exhibit any further difficulties or recurring pre-syncope. No trauma, flu, or recent cat exposure was reported. Family history includes a mother who is the carrier of a hemophilia gene (FXIII), although the child did not carry the gene. Father had a history of epilepsy since age 36 and diagnosed with an auditory neurinoma three years ago. A younger brother aged six and a half years has a partially empty sella turcica. The positive family history for epilepsy initially turned the attention to related causes for the syncope episodes and led to the discovery of the hemangioma.

Best-corrected visual acuity (BCVA) of the left eye was light perception, while the right eye was at 20/20, a sluggish direct reflex of the left pupil was reported, unaffected indirect reflex, normal intraocular pressure, and diffuse retinal edema with a prominent cherry-red spot in the macula. The patient was immediately put on oxygen supply, continuous local massages of the affected eye were performed, acetazolamide treatment was initiated with two 250 mg tablets per os, continuing with one tablet q.d. as well as a 2% dorzolamide hydrochloride single drop t.i.d and trimetazidine dihydrochloride 20 mg t.i.d,, methylprednisolone 20 mg/kg daily, and acetylsalicylic acid 100 mg q.d.

The patient was admitted to the 1st Department of Pediatrics in the hospital in order to treat and diagnose the underlying condition. Neurological examination revealed reduced tendon reflexes in the lower extremities, Romberg (+) test with falling back, difficulty in the finger-to-nose test and walk-in-a-straight-line test, dysdiadochokinesia of the right hand, and reduced muscle tone in the lower extremities. Computed tomography of the brain and intracranial vessels of the neck was performed with contrast and did not reveal any pathology. Blood tests included serum lipoprotein, serum homocysteine ​​and a complete lipid profile, immunological testing and lupus anticoagulants, control for thrombophilia, Hgb electrophoresis, ferritin, and coagulation function, including test for factor V Leiden mutation. MRI, magnetic resonance angiography (MRA), magnetic resonance venography (MRV) of the brain and optical coherence tomography (OCT), optical coherence tomography angiography (OCTA), and fundus photography with pathognomonic findings of the central retinal artery occlusion were performed.

Findings from the OCT

**Figure 1:**
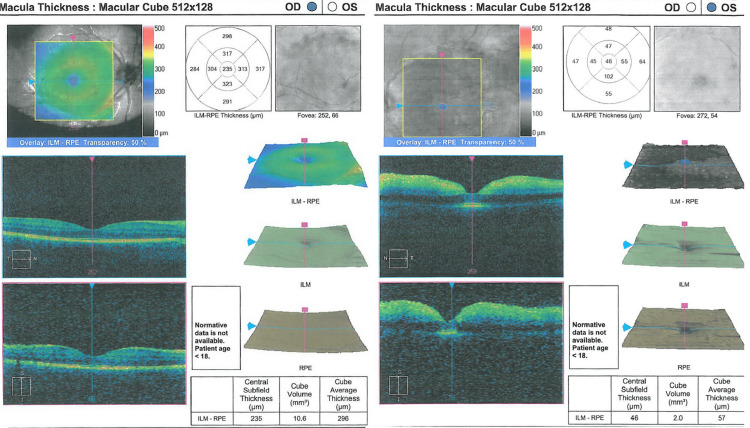
OCT findings from the unaffected right eye (left half of the figure) and of the affected left eye (right half of the figure) OCT of the left eye demonstrates the well-demarcated thickening of the inner retina in the acute phase of arterial occlusion at the macula area that correlates with the areas of swelling of the retina. OCT image at the macula area of the right eye is unremarkable. OCT: optical coherence tomography, OD: oculus dexter (right eye), OS: oculus sinister (left eye), ILM-RPE: internal limiting membrane of the retinal pigment epithelium.

OCT of the left eye demonstrates the well-demarcated thickening of the inner retina in the acute phase of arterial occlusion at the macula area that correlates with the areas of swelling of the retina. OCT image at the macula area of the right eye is unremarkable. Comparative findings of the left and right eye are presented in Figure 1. 

**Figure 2:**
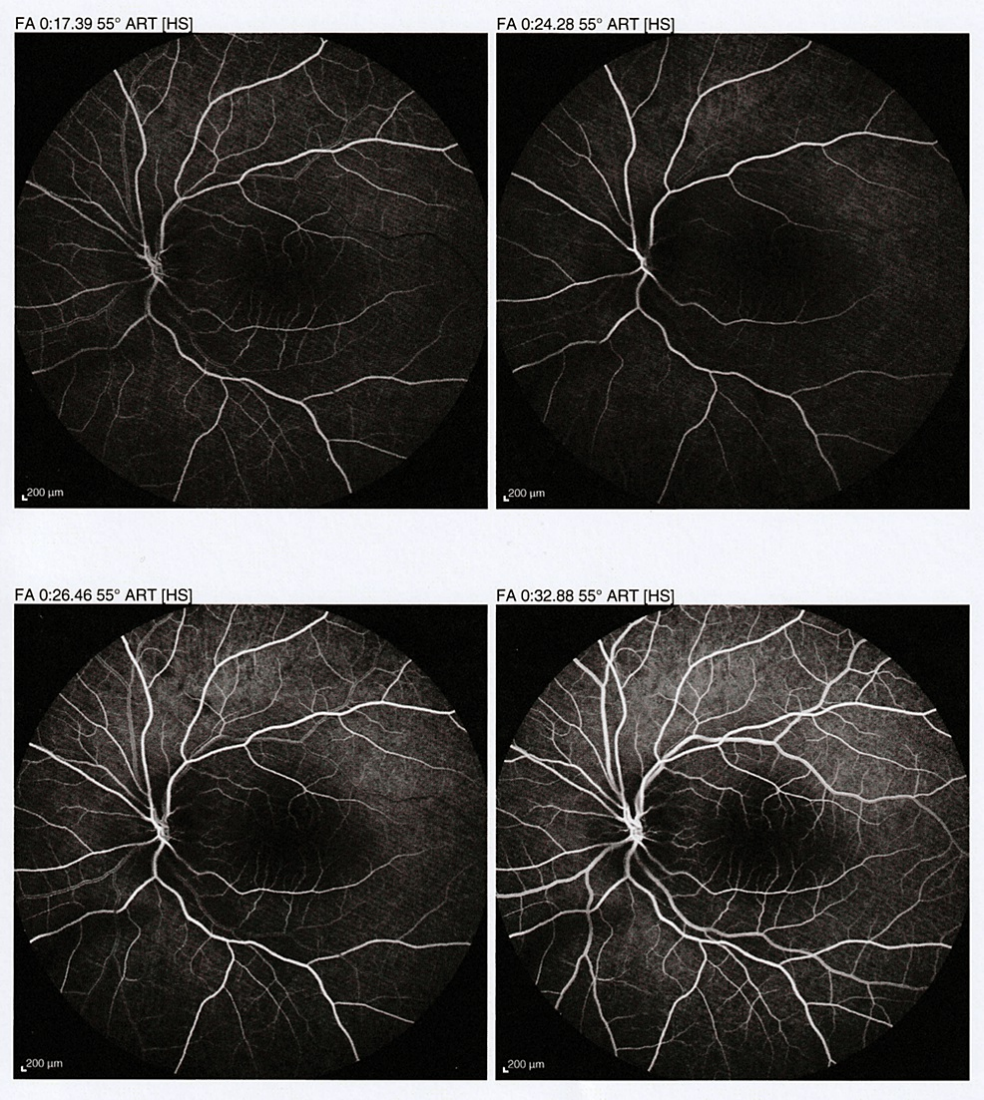
Fluorescein angiography findings of the affected eye Fluorescein angiography findings of the affected eye: four snapshots in time of the fluorescein passage through vascularization of the macula, showing failure to fill in the affected area due to occlusion of the efferent vasculature (central artery) and a hyporeflective area in the outer retinal layers. FA: fluorescein angiography, ART: arm-to-retinal time, HS: high sensitivity.

Fluorescein angiography was performed a week after the event, with no findings from the right eye, while findings from the left eye included the thrombosis of the central artery, hyporeflective area in the outer retinal layers, and deceleration of the vascular fill which completed on 28-30 seconds with arm-to-retinal time (ART) 14 seconds, without any papilledema (Figure 2).

**Figure 3:**
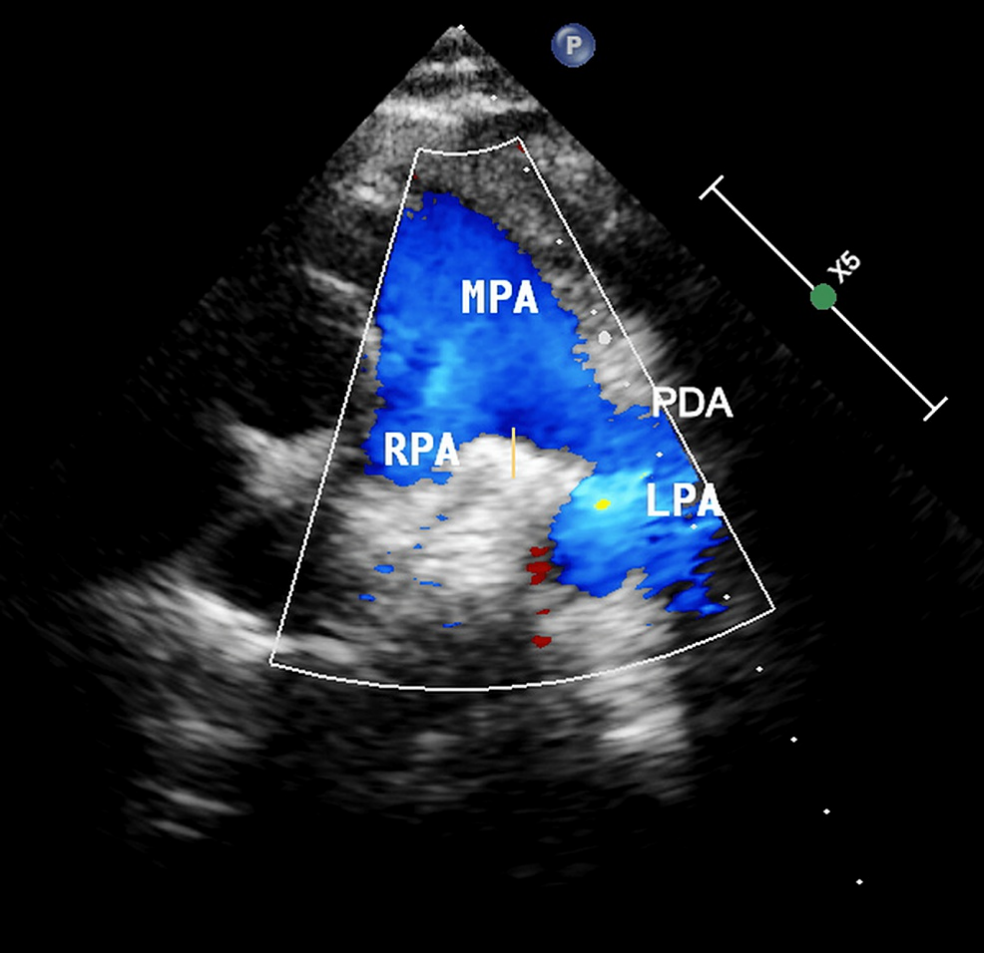
Right-to-left shunt across the patent ductus arteriosus (PDA) MPA, main pulmonary artery; RPA, right pulmonary artery; LPA, left pulmonary artery; PDA, patent ductus arteriosus. A shunt is evident through the PDA.

During the second day of hospitalization, there was a slight improvement of visual acuity (3-4 sc (without correction)) followed from a decrease to <1/10 sc but finally stabilized to 3/10 sc-1.0 cc (with correction). Pediatric cardiology assessment in the Emergency Department (ER) did not reveal pathological findings initially. A transthoracic echocardiogram was performed during the third day of hospitalization and revealed a PDA of approximately 1.5 mm. Short-axis view of the right-to-left shunt across the patent ductus arteriosus is presented in the Doppler echocardiogram (Figure 3). Color Doppler flow pattern is blue during systole and diastole.

The patient’s family were advised to have surgical ligation of the PDA for the prevention of future adverse outcomes.

## Discussion

Despite prompt initiation of symptomatic treatment and initial response, the patient failed to improve. Previous failure to detect the underlying condition, despite the seemingly unexplained fainting episodes, led to a catastrophic outcome. Unfortunately, transthoracic echocardiogram was not performed as part of the protocol for the assessment of syncope episodes. The relative rarity of a PDA in a child born after a full-term pregnancy [2,8] and a lack of clinical signs readily associated with the presence of a PDA should be noted. The paucity of related symptoms in the initial presentation meant that the patient did not receive a potentially life-saving examination. The family history of epilepsy shifted the clinicians' focus in an unfortunate direction and the subsequent absence of a plausible organic cause from a neurological point-of-view further misguided toward a possible psychological explanation. It remains unclear whether these episodes can be attributed to a cerebral underperfusion due to diastolic reverse flow and resulting cerebral hypoxia, a well-known serious issue [1,5], although at this point it appears as a reasonable explanation.

Treatment in the emergency department in similar cases focuses on stabilizing cardiovascular function and limiting the damage induced by the clotting until a surgical solution to the PDA can be administered [1,6]. Unfortunately, damage control was unsuccessful and there are no alternatives that could have been applied; individual patient response cannot be predicted, further highlighting the need for a timely diagnosis. Children are a special needs population since they cannot fully articulate their subjective experience [8]; hence, more detail is needed with physical and diagnostic examinations.

## Conclusions

In conclusion, CRAO occurs rarely in pediatric population in general, but this case highlighted the need to exclude the possibility of an undiagnosed PDA, since even an asymptomatic case could lead to severe outcomes. Clinicians should be aware that rarity does not equate with impossibility. While unnecessary diagnostic procedures should by default be avoided, the lack of a plausible and valid etiology for a serious symptom or clinical presentation does necessitate a thorough follow-up.
